# Application of a Novel Population of Multipotent Stem Cells Derived from Skin Fibroblasts as Donor Cells in Bovine SCNT

**DOI:** 10.1371/journal.pone.0114423

**Published:** 2015-01-20

**Authors:** Shaohui Pan, Wuju Chen, Xu Liu, Jiajia Xiao, Yanqin Wang, Jun Liu, Yue Du, Yongsheng Wang, Yong Zhang

**Affiliations:** 1 College of Veterinary Medicine, Northwest A&F University, Yangling, Shaanxi, China; 2 Key Laboratory of Animal Biotechnology, Ministry of Agriculture, Northwest A&F University, Yangling, Shaanxi, China; UNITED STATES

## Abstract

Undifferentiated stem cells are better donor cells for somatic cell nuclear transfer (SCNT), resulting in more offspring than more differentiated cells. While various stem cell populations have been confirmed to exist in the skin, progress has been restricted due to the lack of a suitable marker for their prospective isolation. To address this fundamental issue, a marker is required that could unambiguously prove the differentiation state of the donor cells. We therefore utilized magnetic activated cell sorting (MACS) to separate a homogeneous population of small SSEA-4^+^ cells from a heterogeneous population of bovine embryonic skin fibroblasts (BEF). SSEA-4^+^ cells were 8-10 μm in diameter and positive for alkaline phosphatase (AP). The percentage of SSEA-4^+^ cells within the cultured BEF population was low (2-3%). Immunocytochemistry and PCR analyses revealed that SSEA-4^+^ cells expressed pluripotency-related markers, and could differentiate into cells comprising all three germ layers in vitro. They remained undifferentiated over 20 passages in suspension culture. In addition, cloned embryos derived from SSEA-4 cells showed significant differences in cleavage rate and blastocyst development when compared with those from BEF and SSEA-4^−^ cells. Moreover, blastocysts derived from SSEA-4^+^ cells showed a higher total cell number and lower apoptotic index as compared to BEF and SSEA-4^–^ derived cells. It is well known that nuclei from pluripotent stem cells yield a higher cloning efficiency than those from adult somatic cells, however, pluripotent stem cells are relatively difficult to obtain from bovine. The SSEA-4^+^ cells described in the current study provide an attractive candidate for SCNT and a promising platform for the generation of transgenic cattle.

## Introduction

Since Dolly’s birth, many mammalian species have been sucessfully cloned by SCNT. SCNT is a cloning technique in which the nucleus from a somatic donor cell is inserted into an enucleated oocyte to create a viable embryo that is then implanted into a host animal for reproduction. Despite major efforts in the last decade to improve this technology [1], the total efficiency remains low and cloning animals by SCNT is generally inefficient. Many factors have been reported to influence the total efficiency of this technology, of which nuclear donor cells is a crucial factor. Tissue specificity, cell type, age, status, and the cell cycle of donor cells affected the development of cloned embryos [2]. The cloning from fully differentiated somatic cells appears to be extremely inefficient [3]. It has been suggested that less differentiated cells may be more amenable to nuclear transplantation (NT) than terminally differentiated cells, as stem-like cells may possess the developmental plasticity required for proper reprogramming, thus making them a better candidate for SCNT [4–6].

Adult stem cells are unique populations of undifferentiated cells within various tissues that have a high capacity for self-renewal. Several studies have shown that various stem cell populations within one tissue can give rise to differentiated cell types of other tissues across multiple embryonic lineages [7–10], which is a process known as transdifferentiation. Therefore, these types of stem cells from the tissue microenvironment may have significantly more capacity for transdifferentiation and ultimately for the purpose of reprogramming by SCNT. Several groups of investigators have reported the presence of multipotent stem cells in adult tissues [11–14]. Stage specific embryonic antigen-4 (SSEA-4) which is a cell surface marker detected in pluripotent cells has previously been used as a marker to isolate novel stem cell subpopulations from human bone marrow [15, 16], human pancreas [17], human dermis [18] and other tissues [19–21]. Specifically, multipotent stem cells have been isolated from the mouse dermis that can form neural and adipose cells [7], thereby confirming the existence of progenitors in the skin that have a high capacity for differentiation into multiple cell types. Adult skin stem cells are useful for studying development and differentiation. To repair damaged tissue, the skin depends on stem cell populations residing in the adult hair follicle, sebaceous gland, dermis and epidermis for continuous self-renewal [22]. Recently, several studies have identified a subpopulation of stem-like cells in human dermal fibroblasts [23, 24]. These cells expressed pluripotency markers and were able to differentiate into endodermal, ectodermal, and mesodermal cells. Furthermore, these cells showed enhanced efficiency in generating induced pluripotent stem (iPS) cells. These subpopulations of multipotent stem cells from farm animals are valuable cell models for the study of development, differentiation, and are potential efficient donors for NT. These types of multipotent progenitors have also been isolated from the skin of various farm animals, and may ultimately provide a source of efficient donor cells for SCNT. For example, stem cells isolated from porcine skin demonstrated multilineage potential, expressing the neural progenitor marker, nestin, as well as genes that are critical for pluripotency such as Oct4 [25]. These porcine skin stem cells also had the intrinsic ability to differentiate into oocyte-like cells [26]. Furthermore, a subpopulation of multipotent stem cells derived from goat skin enhanced the development and quality of cloned goat embryos in vitro [27, 28]. While these studies demonstrate the potential of these multipotent stem cells in improving cloning strategies, further work needs to be done to fully characterize these cells in order to maximize cloning efficacy and to streamline the process of transgenic animal production.

Pluripotent stem cells from domesticated animals have potential applications in transgenic animal production. Cattle are the most common type of large, domesticated ungulates. To date, stable embryonic stem (ES) cell lines have not been generated from bovine embryos. Although iPS technology has been used to generate bovine iPS cells with defining features similar to either human or mouse ES cells [29, 30], several important issues remain to be addressed, including the low efficacy of generating fully reprogrammed bovine iPS cell lines as well as the safety of iPS cells. SSEA-4^+^ cells derived from domesticated animals have not been reported. In the current study, we report the isolation and characterization of bovine small SSEA-4^+^ cells from the skin by MACS. Furthermore, we demonstrate that the isolated cells expressed pluripotency markers and were able to differentiate into endodermal, ectodermal, and mesodermal cells. After SCNT, embryos cloned from multipotent SSEA-4^+^ cells show higher developmental competence in vitro, demonstrating that SSEA-4 can be used as a marker to enhance efficiency of bovine SCNT.

## Materials and Methods

### Ethics statement

All animals were handled in strict accordance with good animal practices as defined by the relevant national and/or local animal welfare bodies. The experimental procedure was approved by the Animal Care and Use Committee of Northwest A&F University, China, and performed in accordance with animal welfare and ethics guidelines. Mice were killed by cervical dislocation, and all efforts were made to minimize animal pain and suffering.

### Cell isolation and culture

Bovine fetal skin tissue was excised from the back of a healthy 90-day-old fetus. The fetus was obtained following a spontaneous abortion recovered from a startled cow housed at the animal experiment center of the university. Fetal skin tissue samples (8 total) were then cut into 1 mm^3^ pieces. The pieces were washed three times in Ca^2+^/Mg^2+^ free Phosphate Buffered Saline (PBS) and digested with 0.25% trypsin for 35 min at 37°C, followed by 0.1% DNase for 5 min at room temperature. Tissue pieces were washed with PBS two times, and mechanically dissociated by vortexing and pipetting with 2 ml medium. The cell suspension was passed through a 40 μm strainer (Falcon), centrifuged, and resuspended in DMEMF12(1:1) supplemented with antibiotics, 10% fetal bovine serum (FBS) (Hyclone, Logan, Utah, USA), B-27 (Gibco), 20 ng/ml EGF (Sigma), and 40 ng/ml bFGF (Sigma). Cells were cultured on 60 mm tissue culture dishes in a 37°C, 5% CO_2_ tissue-culture incubator. Culture medium was changed every 2–3 days. Upon reaching 80–90% confluence, cells were harvested by trypsinization (0.05% trypsin and 2 mM EDTA). Primary bovine embryonic fibroblasts were passaged at a ratio of 1:3 or frozen in 10% dimethylsulfoxide (DMSO), 90% Dulbecco’s modified Eagle medium/F12 (DMEM/F12) supplemented with 10% FBS.

### Isolation of SSEA-4^+^ cells by Magnetic Activated Cell Sorting (MACS)

For cell sorting, fibroblasts at first passage (P1) were dissociated to a single-cell suspension by pipetting up and down and passed through 30 μm nylon mesh to remove cell clumps which may clog the column. Cells were centrifuged at 300 g for 5 min and 10^7^ total cells were resuspended in 80 μl of ice-cold buffer (DPBS without Ca^2+^ and Mg^2+^, with 0.5% bovine serum albumin, and 2 mM EDTA ). 20 μl of Anti-SSEA-4 Micro Beads (Miltenyi Biotec) was added per 10^7^ total cells, incubated for 15 min in the refrigerator (2–8°C). Cells were washed with 1–2 ml of buffer per 10^7^ cells, centrifuged at 300 g for 5 min, and resuspended in 500 μl of buffer. A MACS column was placed in the magnetic field of a suitable MACS Separator and prepared by rinsing with 3 ml of buffer. The column was washed three times with 3 ml of buffer. Flow-through of unlabeled cells (SSEA-4^−^ cells) were collected. The column was placed over a collection tube, 5 ml buffer was pipetted onto the column, and the magnetically labeled cells (SSEA-4^+^ cells) were flushed out by firmly pushing the plunger into the column. SSEA-4^+^ and SSEA-4^−^ cells were cultured under suspension conditions.

### Suspension Culture

Cells were seeded onto 24-well Ultra-Low Attachment cell culture plate (Corning), at a concentration of 5000 cells/ml, and cultured in DMEMF12(1:1) supplemented with antibiotics, B-27 (Gibco), 20 ng/ml EGF (Sigma), 40 ng/ml bFGF (Sigma). A volume equal to one third of the initial culture medium was added to the dishes every 2 days, and after 7 days in culture, cell clusters were collected, dissociated using 0.25% trypsin for 15 min, and returned to suspension culture.

### Alkaline Phosphatase Staining

The SSEA-4^+^ cells and cell clusters derived from suspension culture were washed at least three times with PBS. Staining was performed at room temperature in the dark using the 5-bromo-4-chloro-3-indoly phosphate/nitro-blue tetrazolium (BCIP/NBT) Alkaline Phosphatase Color Development Kit (Beyotime, Jiangsu, PR China) according to the manufacturer’s instructions.

### Immunofluorescence Staining

Cells were were fixed in 4% paraformaldehyde (PFA) for 15 min, permeabilized with 0.1% Triton X-100 for 30 min, and then incubated with blocking solution (PBS + 1% BSA) at 4°C for 30 min followed by primary antibodies at 4°C overnight. Primary antibodies were diluted in Immnol Fluorence Staining Primary Antibody Dilution Solution (Beyotime, P0108) at the followings ratios: OCT4 (rabbit polyclonal antibody, 1:500, Abcam), SOX2 (rabbit polyclonal antibody, 1:500, Abcam), NANOG (rabbit polyclonal antibody, 1:500, Abcam), SSEA4 (rabbit polyclonal antibody, 1:500, Abcam), CD105 (rabbit monoclonal antibody, 1:500, Abcam), β-Ⅲ-tubulin (rabbit monoclonal antibody, 1:500, Chemicon), AFP (mouse monoclonal antibody, 1:500, Chemicon), Desmin (mouse polyclonal antibody, 1:500, Abcam), Nestin (rabbit monoclonal antibody, 1:200, Chemicon), PDX1 (rabbit monoclonal antibody, 1:500, Chemicon) and α-actin (rabbit monoclonal antibody, 1:200, Sigma). After washing three times with PBS, cells were incubated with Alexa Fluor 488 (donkey anti-mouse) or Alexa Fluor 546 (goat anti-rabbit) conjugated secondary antibody (1:500; Chemicon) for 1h at room temperature in the dark. Finally, DNA was stained with Hoechst33342 (Beyotime, C1005) for 3 min. Negative controls were processed the same way, except that the primary antibodies were replaced with blocking buffer.

### Flow Cytometric Analysis

The DNA content and surface antigens of cells were examined by flow cytometry analyses. To detect DNA content, cells were dispersed, collected, washed twice with PBS, and fixed with 1 ml of 70% of ice-cold ethanol at 4°C for at least 12 h. Cells were then washed twice with PBS, resuspended in 0.5 ml PBS, with propidium iodide (PI) at a final concentration of 50 mg/ml and RNaseA added, incubated for 30 min at 37 ºC. Cells were stained with fluorescein isothiocyanate (FITC)-conjugated or phycoerythrin(PE)-conjugated antibodies (Becton Dickinson) against the markers commonly used to define mesenchymal stromal cells (MSCs), CD44, CD71, CD29, CD166, CD105 and CD34, according the manufacturers’ instructions. To detect the expression of Oct4, cells were detached and stained with Anti-Oct4 (Alexa Flour 488 conjugate). Cells were analyzed using a FACS Calibur flow cytometer (Beckman, Brea, CA, USA). The primary antibodies were listed in Table 1.

**Table 1:** Primary Antibodies for Flow Cytometry.

**Antigen**	**Clone**	**Conjugation**	**Isotype**	**Supplier**
IgG1	G18–145	FITC	Ms monoclonal IgG1,k	BD Pharmingen
CD44	G44–26	FITC	Ms monoclonal IgG2b, κ	BD Pharmingen
CD29	Ha2/5	FITC	Ar Ham monoclonal IgM, l	BD Pharmingen
CD71	266	FITC	Ms monoclonal IgG1, o	BD Pharmingen
IgG1	MOPC-21	PE	Ms monoclonal IgG1, o	BD Pharmingen
CD166	3A6	PE	Ms monoclonal IgG1, κ	BD Pharmingen
CD105	266	PE	Ms monoclonal IgG1, κ	BD Pharmingen
CD34	8G12	PE	Ms monoclonal IgG1, κ	BD Biosciences
Oct4	10H11.2	Alexa Flour 488	Human monoclonal IgG1	Millipore

### BrdU incorporation assay

The proliferation of BEF and SSEA-4^+^ cells were assayed by BrdU incorporation, performed similarly to previous report [31]. Firstly, cells were treated with 30 mg/mL BrdU (Sigma, St Louis, MO, USA) for 6 h, then subjected to BrdU immunostaining. More specifically, cells were fixed in 4% PFA for 15 min at room temperature and washed three times, for 10 min each with PBS (pH 7.4) containing 0.1% Triton X-100. The cells were then washed three times in PBS (pH 7.4) alone. Anti-BrdU (1:100; Santa Cruz) dissolved in 0.1 M PBS (pH 7.4) containing 5% normal goat serum was added, and cells were incubated overnight at 4°C. Cells were washed in PBS (pH 7.4) three times, then incubated with secondary antibody (FITC, Millipore 1:500) for 1 h at room temperature. Three more washes were carried out, and cells were visualized under a Leica fluorescent microscope and analyzed for BrdU uptake. The percentage of BrdU-positive cells was calculated by manual counting.

### RNA extraction and RT-PCR

Total RNA was extracted from each sample using RNAprep Pure Micro Kit (Qiagen) according to the manufacturer’s instructions. First strand cDNAs were prepared from 0.5 μg RNA using PrimeScript RT reagent Kit (TaKaRa, Dalian, China) and gene expression was analyzed. PCR conditions were as follows: initial denaturation at 94°C for 5 min, followed by 30 cycles of 94°C for 30 s, the annealing temperature used was in accordance with the primer sequence for 30 s and 72°C for 30 s with a final extension at 72°C for 10 min. The PCR products were analyzed in 2% agarose (Invitrogen) by gel electrophoresis, stained with ethidium bromide (Invitrogen), and visualized under UV illumination. The primer sequences were designed cross-intron, and PCR amplification product sizes are described in Table 2.

**Table 2:** The Primer Sequences and PCR Reaction Conditions.

**Gene**	**Sequence (5’- 3’)**	**Tm/°C**	**Size/bp**	**Accession number**
β-actin	F: GCGGCATCCACGAAACTAC	58	138	EU655628
	R: TGATCTCCTTCTGCATCCTGTC			
OCT4	F: CCACACTCGGACCACGTCTT	58	187	NC_007324.5
	R: GCACCAGGGTCTCCGATTT			
SOX2	F: CTATGACCAGCTCGCAGA	54	152	NC_007299.5
	R: GGAAGAAGAGGTAACCACG			
NANOG	F: GATTCTTCTACCAGTCCCAAAC	54	176	NC_007303.5
	R: CTGTCTCTCCTCTTCCCTCCTC			
C-MYC	F: CACGCTGACCAAGGTAT	58	220	NC_007307.5
	R: CTGAGGTGGTTCATACTGA			
AFP	F: TGCCCGATGATAAGGT	58	306	NM_214317.1
	R: TACTCGGAACCTCACTTTTGTC			
Brachury	F: AAGAACGGCAGGAGGATGG	58	372	XM_001928144.1
	R: CTCTGGGAAGCAGTGGC			
FGF5	F: TCACGGGGAGAAGCGTCT	54	407	NM_004464.3
	R: ACTTGGCACTTGCATGGA			
PPARγ	F: GATGCCACAGGCCGAGAAGG	58	235	NC_000003.10
	R: GCCCTGAAAGATGCGGATGG			
GATA4	F: CTGTGCCAACTGCCAGACCA	58	437	NM_214293.1
	R: GGCTGACCGAAGATGCGTAG			

### qRT-PCR

Real-time PCR was performed using SYBR Premix Ex TaqTM (TaKaRa) and a StepOne Plus thermocycler (Applied Biosystems). The reactions were set up in 25 µl reaction mixtures containing 12.5 μl 1×SYBR Premix Ex TaqTM (TaKaRa), 0.5 μl sense primer (10 μM), 0.5 μl antisense primer (10 μM), 11 μl distilled water, and 0.5 μl template ( 100 ng/μl). The reaction conditions were as follows: 95°C for 10 sec, followed by 40 cycles at 95°C for 5 sec and 60°C for 30 sec. The β-actin gene was used as a reference gene. For each sample, target and reference genes were amplified independently on the same plate and in the same experimental run in triplicate. PCR specificity was confirmed by gel electrophoresis on a 2.5% agarose gel, and by a single peak in the melting curve. The 2^–ΔΔCT^ method was used to quantify the relative mRNA levels. In this test, real-time PCR assays were repeated three times. Fold changes were then compared using one-way ANOVA followed by Newman-Keuls test.

### Karyotype analysis

Chromosomes were analyzed in actively proliferating cultures of SSEA-4^+^ cells. Karyotype analysis was performed using the method reported by He et al. [32].

### In vitro differentiation

To induce SSEA-4^+^ cells to form cells of the three germ layer lineages, SSEA-4^+^ cells at P8 were harvested with 0.25% Trypsin and reseeded in ultra-low binding dishes at a concentration of 1×10^6^ cells/ml to form cell clusters for 3 days. The cell clusters were then transferred to gelatin-coated 24-well culture tissue plates and grown in the induction media to induce cell differentiation. BEF cultured in induction medium were used as negative controls. For neural induction, cells were cultured in Neurobasal medium supplemented with B-27 supplement, 30 ng/ml basic fibroblast growth factor (bFGF; PeproTech Inc., Rocky Hill, NJ, USA), and 30 ng/ml epidermal growth factor (EGF; PeproTech). The neural stem cell spheres were subjected to induction regimes for further differentiation. For cardiac induction, expanded cells were cultured on gelatin-coated dishes in DMEM containing 10% FBS, 0.1 mM Vc (Sigma), 10 mM 5-aza (Sigma) for 14–21 days with culture medium changed every 2 days. For pancreatic cells induction, cells were cultured in RPMI 1640/B27 medium supplemented with 1% BSA, 100 mM nicotinamide (NIC, Sigma), 10 ng/ml exendin-4 (Sigma), 1 mM sodium pyruvate (Invitrogen), 2 mM glutamine (Invitrogen), 1 mM β-mercaptoethanol (Sigma), 20 ng/ml EGF and 20 ng/ml bFGF for 7 days. Cells were then cultured for another 7 days in the same medium supplemented with 10^-6^ M RA (Sigma) but without EGF and bFGF. The medium was changed every 3 days. After the induction, the tissue specific gene markers were then examined to determine the level of the differentiation by immunocytochemistry and RT-PCR analysis.

To determine whether SSEA-4^+^ cells at P3 exhibited differentiation potential similar to that of mesenchymal stem cells, we used the Differentiation Kits (Cyagen Biosciences Inc., Guangzhou, China), according to manufacturer’s instructions. To perform adipogenic, osteogenic and chondrogenic differentiation, cells were expanded in either adipogenic, osteogenic or chondrogenic differentiation media (Cyagen Biosciences) in 6-well plates. Adipogenic, osteogenic and chondrogenic differentiation was then assayed using the standard protocol from Cyagen Biosciences Inc.

### Teratoma formation

SSEA-4^+^ cells at P3 were harvested and dissociated to a single-cell suspension. Three 4-week-old (BALB/c nu/nu) mice purchased from the Shanghai Institute of Tumor Research (Shanghai, China) were housed under specific pathogen-free conditions. Cells (5×10^6^ cells/injection) were subcutaneously injected into the right axilla of three nude mice. As positive and negative controls, J1 mESCs and BEF at the same concentration were injected into the right axilla of three other nude mice. The mice were observed weekly for 5–8 weeks.

### Nuclear transfer

NT was performed as previously described [28]. BEF, SSEA-4^+^ and SSEA-4^−^ cells at passages 1–3 were used as donor cells for SCNT to investigate the in vitro development of cloned embryos. Embryos were cultured for 7 days to evaluate their developmental rate.

### Differential staining and apoptosis assay of cloned blastocysts

Differential staining and apoptosis assay were carried out in accordance with the methods of our previous study. After 7 days of culture in vitro, cloned blastocysts from BEF, SSEA-4^+^ and SSEA-4^−^ cells were used for differential staining and apoptosis assay. Blastocyst cell numbers were estimated by counting the total number of nuclei using DAPI, whereas the number of trophectoderm (TE) cells was estimated using immunostaining for CDX2. The number of inner cell mass (ICM) cells was determined as the total number of nuclei minus the number of TE nuclei. Apoptosis was investigated using the Dead End Fluorometric TUNEL System (Promega, Madison, WI, USA).

### Statistical analysis

Using SPSS18.0 statistical software (IBM Corporation, Somers, NY, USA), all data were analyzed by one-way ANOVA and LSD tests, and reported as mean ± SEM. For all analyses, P < 0.05 was considered statistically significant.

## Results

### Isolation and culture of bovine embryonic skin fibroblasts

**Figure 1:**
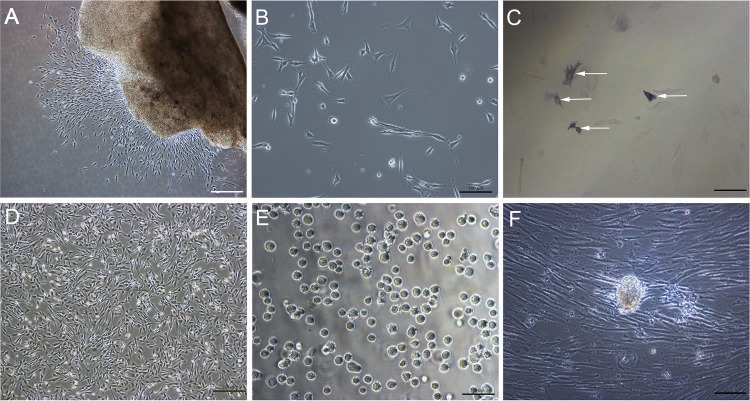
Culture of bovine embryonic fibroblasts. (A) Outgrowth of fibroblasts from a section of fetal bovine skin. (B) Bovine embryonic fibroblasts demonstrating a heterogeneous population of cells. (C) AP-positive cells within the population of bovine embryonic fibroblasts. (D) Several floating cells growing on top of fibroblasts. (E) Several cells displaying a rough surface with a long cilium. (F) Cell clusters that occur spontaneously in adherent cultures of naive skin fibroblasts. A-D, F: Scale bar = 100 *μ*m; E: Scale bar = 50 *μ*m.

BEF were isolated from bovine fetal skin by allowing the tissue explant to adhere, and grow out in culture. BEF (Fig. 1A) showed the first outgrowth from skin biopsies on day 2–3, and reached 70–80% confluence by day 7. We observed that naive BEF consisted of a heterogeneous population (Fig. 1B). The majority of cells exhibited a typical spindle-shaped morphology, while the remaining cells were small with a non-fibroblastic phenotype, and stained positive for AP (Fig. 1C). In culturing the cells on plastic, several floating cells grew on top of the adherent fibroblasts. The surface of these cells was rough with a long cilium. Naive BEF cultured under adherent conditions spontaneously formed cell clusters at a very low frequency, that appeared similar to clusters formed by early stage ES cells (Fig. 1F), suggesting that the heterogeneous population of BEF may contain multipotent stem cells.

### Isolation and culture of SSEA-4^+^ cells

Upon reaching 70–80% confluence, BEF at P1 were prepared for isolating SSEA-4^+^ cells by MACS as described in the methods section above. Approximately 2–3% cells of naive BEF expressed SSEA-4, therefore the quantity of primary SSEA-4^+^ cells isolated from BEF was relatively low. However, the percentage of SSEA-4^+^ cells in the total BEF population was affected by cell passage. After sorting and collecting the SSEA-4^+^ cells, both SSEA-4^+^ and SSEA-4^−^ cells were cultured under adherent conditions, as described previously in the methods section. The cells were capable of growing to 60–80% confluence by day 7. SSEA-4^+^ cells underwent growth arrest after 20 passages while BEF could be subcultured for up to 40 passages under suspension conditions. This result suggested that SSEA-4^+^ cells were not suitable for long term adherent cell culture. Consequently, cells were transferred to ultra-low-attachment dishes and cultured in suspension to maintain the undifferentiated state. The cell clusters formed in suspension were separated into single cells by trypsin/EDTA and transferred to ultra-low-attachment dishes.

### Characterization of SSEA-4^+^ cells

**Figure 2:**
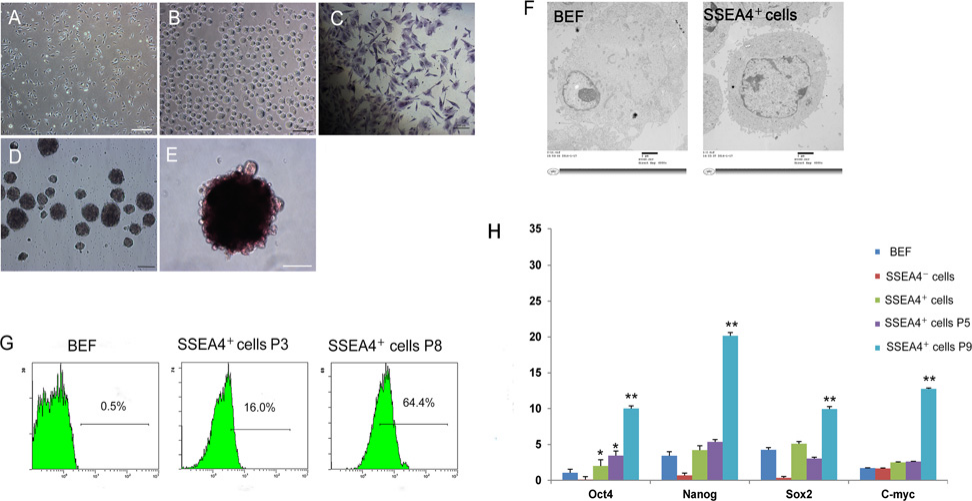
Isolation of SSEA-4^+^ cells. (A) SSEA-4^+^ cells at P1, were isolated from bovine fetal skin fibroblasts by MACS. (B) SSEA-4^+^ cells demonstrate a rough surface and a long cilium. (C) Positive AP staining of SSEA-4^+^ cells. (D) Floating spheres of SSEA-4^+^ cells in suspension culture for 3 days. (E) AP staining of cell clusters from SSEA-4^+^ cells. (F) TEM of BEF and SSEA-4^+^ cells. (G) Flow cytometry analysis exhibiting the percentage of BEF, SSEA-4^+^ cells at P3, and SSEA-4^+^ cells at P8 that expressed Oct4. (H) qRT-PCR analysis of OCT4, NANOG, SOX2, and C-MYC gene expression in BEF, SSEA-4^−^ cells, SSEA-4^+^ cells at P1, SSEA-4^+^ cells at P5, and SSEA-4^+^ cells at P9. All data are presented as the mean±SEM and are derived from three independent experiments (*P<0.05; **P<0.01). A: Scale bar = 100 *μ*m; B-E: Scale bar = 50 *μ*m.

**Figure 3:**
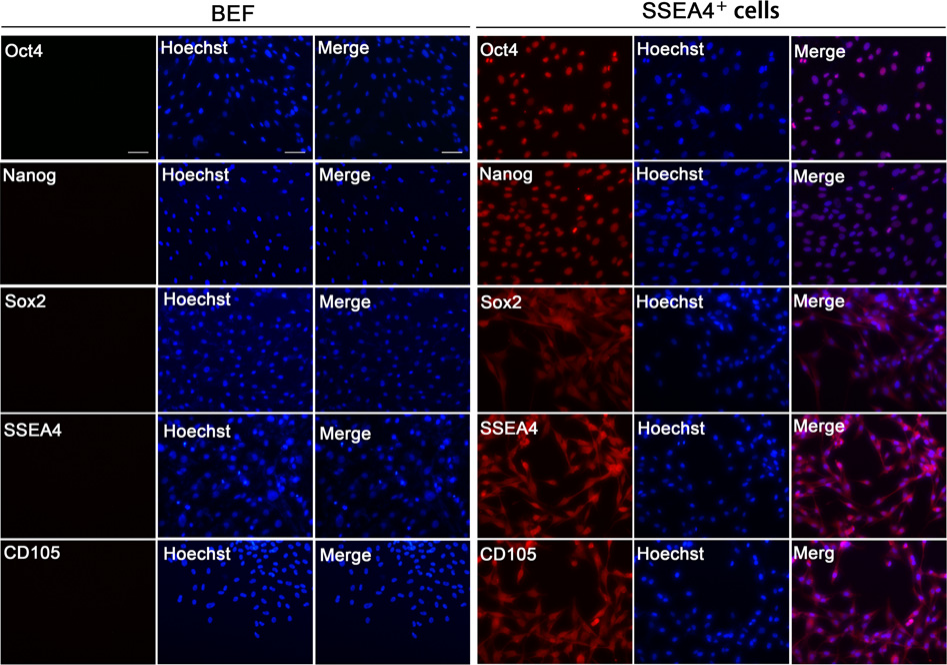
Immunofluorescence analysis of SSEA-4^+^ cells. OCT4, NANOG, SOX2, SSEA4 and CD105 primary antibodies were detected with Alexa Fluor 555 (red); DNA was stained with Hoechst 33342 (blue). Scale bar = 50 μm.

**Figure 4:**
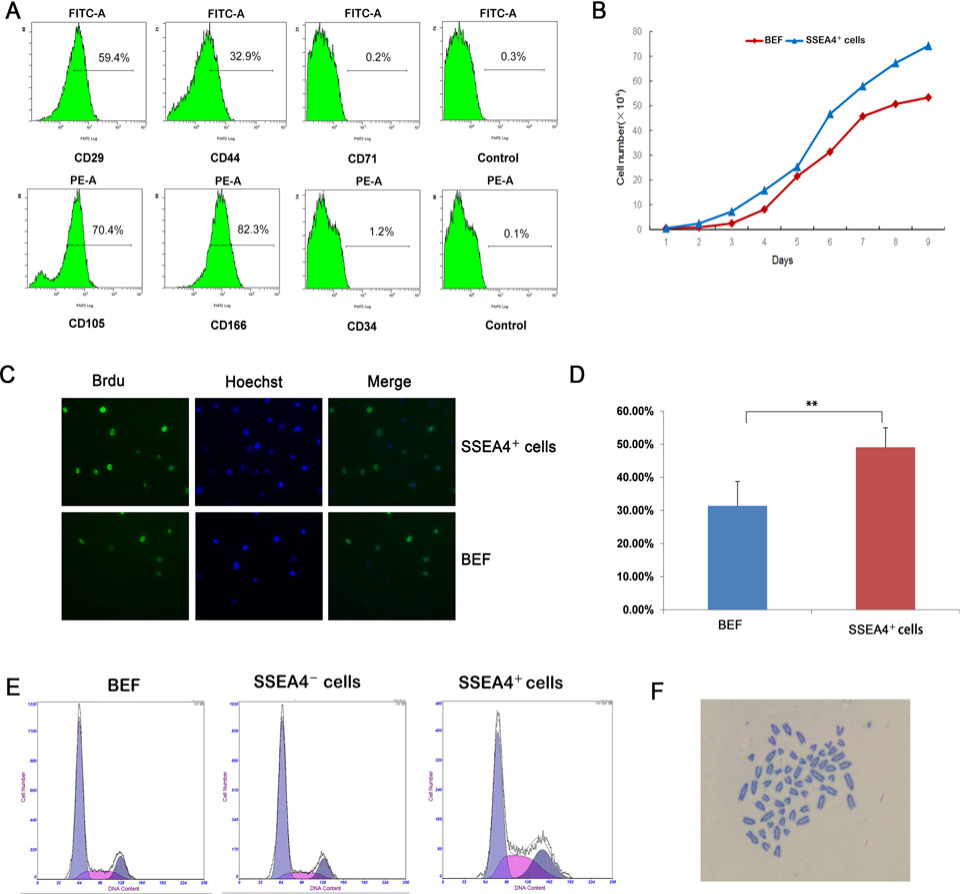
Characterization of SSEA-4^+^ cells. (A) Flow cytometry analysis demonstrating that SSEA-4^+^ cells were positive for mesenchymal markers, CD166, CD105, CD29, and CD44 and negative for hematopoiesis-related markers, CD34, CD71. (B) Cell proliferation curve of BEF and SSEA-4^+^ cells at P3. (C) The mitotic index of SSEA-4^+^ cells was significantly higher than that of BEF. (D) The percentage of BrdU positive cells of SSEA-4^+^ cells was significantly higher than that of BEF(** p<0.01). (E) Cell cycle analysis of BEF, SSEA-4^+^ and SSEA-4^−^ cells at P3. (F) Karyotype analysis of SSEA-4^+^ cells at P20.

SSEA-4^+^ cells were small and round with diameters of 8–10 μm (Fig. 2A-B), displayed a rough surface with a long flagellum, and the majority were positive for AP staining (Fig. 2C). They formed cell clusters at 3 days in suspension, which grew to a diameter about 50–100 μm by day 7 (Fig. 2D). Cell clusters formed from SSEA-4^+^ cells stained positive for AP (Fig. 2E), and the nucleus of SSEA-4^+^ cells was relatively large (Fig. 2F). In contrast, SSEA-4^−^ cells and BEF formed only a few cell clusters with 50 μm in diameter even after 7 days in suspension. Immunofluorescence analysis showed that cell clusters from SSEA-4^+^ cells were consistently positive for the pluripotent markers NANOG, OCT4, SOX2, SSEA-4 and CD105 (Fig. 3). Analysis by flow cytometry further showed that 0.5%, 16.0%, and 64.4% of BEF, SSEA-4^+^ cells (P3), and SSEA-4^+^ cells (P8), respectively, expressed OCT4 (Fig. 2G). qRT-PCR analysis for gene expression of OCT4 demonstrated that SSEA-4^+^ cells expressed higher levels of the pluripotent marker than BEF and SSEA-4^−^ cells (Fig. 2H). Furthermore, there was an increase in the expression of these pluripotent stem cell markers with increased passage of these cells. SSEA-4^+^ cells were also positive for mesenchymal markers, CD166, CD105, CD29 and CD44 and negative for hematopoiesis-related markers, CD34 and CD71, as determined by flow cytometry (Fig. 4A). The SSEA-4^+^ cells at P20 displayed a normal karyotype containing 60 chromosomes (2n = 60) with no acquired numerical or structural aberrations or chromosome/chromatid breaks (Fig. 4F).

### Proliferation potential of SSEA-4^+^ cells

To determine the proliferative capacity of SSEA-4^+^ cells, we subcultured them in parallel with BEF for several months, and discovered that SSEA-4^+^ cells maintained a relatively higher growth rate than BEF, with cells being passaged every 2 to 3 days. Cell proliferation, determined by a growth curve assay, showed that the SSEA-4^+^ cells proliferated rapidly in vitro, multiplying nearly 140-times in 9 days of suspension culture (Fig. 4B). The mean population doubling time (PDT) of SSEA-4^+^ cells (P5) was 27.2 h as compared to a PDT of 32.4 h for BEF (P8). BrdU incorporation assay demonstrated that the mitotic index of SSEA-4^+^ cells (49.06%) was significantly higher than that of BEF (31.37%) (Fig. 4E-D).

### Differentiation potential of SSEA-4^+^ cells

**Figure 5:**
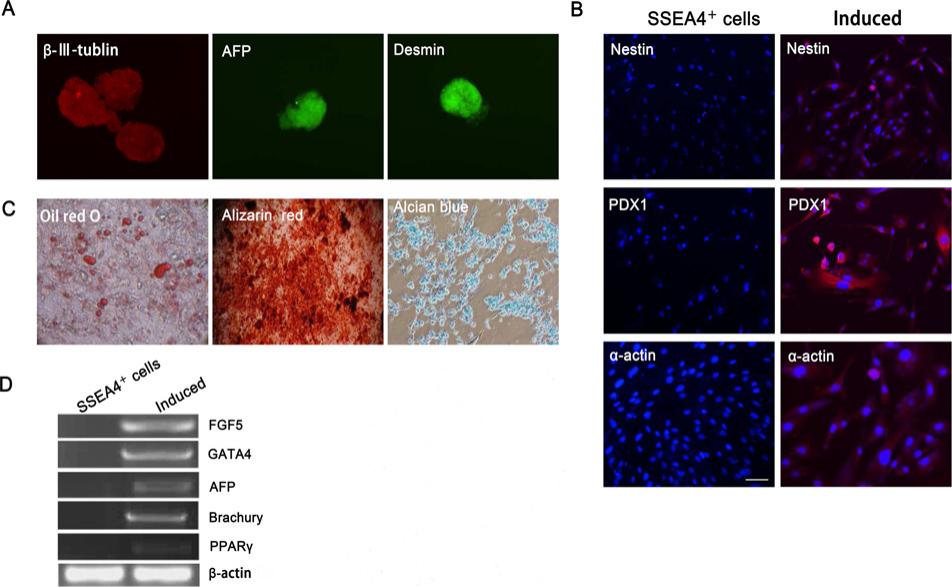
The differentiation potential of SSEA-4^+^ cells in vitro. (A) The floating spheres derived from SSEA-4^+^ cells spontaneously differentiated into the three types of germ layers expressing specific markers for each, including β-III-tubulin (ectoderm), AFP (endoderm) and Desmin (mesoderm). (B) SSEA-4^+^ cells differentiated into specific cells as determined by immunofluorescence analysis for Nestin, PDX1, and α-actin expression, respectively. (C) In vitro differentiation of SSEA-4＋ cells into adipocytes, osteocytes, and chondrocytes assayed using Oil red O stain for adipogenesis, Alizarin Red stain for osteogenesis, and Alcian blue stain for chondrogenesis. (D) RT-PCR assay for specific markers of the three germ layers including FGF5, GATA4, Brachury, AFP, and PPARγ. β-actin was used as the internal control. Scale bar = 50 μm.

To assess the ability to differentiate in vitro, cell clusters formed by SSEA-4^+^ cells at P8 were transferred onto gelatin-coated dishes. The cell clusters spontaneously differentiated into the three types of germ layers expressing specific markers for each, including β-III-tubulin (ectoderm), AFP (endoderm), and Desmin (mesoderm) (Fig. 5A). Immunofluorescence analysis was performed to determine Nestin, PDX1, and α-actin expression. Results revealed that differentiated cells were positive for the neural stem cell marker, Nestin, the pancreatic stem cell marker, PDX1, and the cardiomyocyte cell marker, α-actin (Fig. 5B). RT-PCR analysis also confirmed that induced cells expressed markers specific for the three germ layers including the ectodermal marker, FGF5, the mesodermal markers, Brachury and PPARγ, and the endodermal markers, GATA4 and AFP (Fig. 5D). However, these markers were not detected in bovine fibroblasts nor bovine fibroblasts cultured in various types of differentiation media demonstrating their inability to differentiate into cells from the three germ layers.

To further address the differentiation potential of SSEA-4^+^ cells, cells were cultured under appropriate conditions to induce adipocyte, osteocyblast, and chondrocyte differentiation. In adipogenic induction medium, lipid droplets appeared in the cytoplasm after one week and were positive for Oil red O. When cultured under osteogenic conditions, SSEA-4^+^ cells acquired an osteoblastic phenotype with deposition of a calcium-rich, mineralized extracellular matrix, as revealed by Alizarin Red stain. SSEA-4^+^ cells cultured as pellets under chondrogenic conditions stained positive for Alcian blue (Fig. 5C). We injected SSEA-4^+^ cells subcutaneously into the right axilla of three immunodeficient mice, and observed no teratoma formation in any of the mice at 12 weeks post-injection. The positive control (J1 mESCs) formed teratomas.

### Development of cloned embryos in vitro

Cloned embryos from BEF and SSEA-4^−^ cells were used as controls. There was no difference in fusion rate among the embryos derived from the three cell types (78.19±1.31%, 73.31±1.74%, and 73.92±1.38% respectively, P>0.05) (Table 3). Cloned embryos from SSEA-4^−^ cells showed significantly lower cleavage rates when compared with those from SSEA-4^+^ cells or BEF (66.32±1.47% vs 85.62±1.16% and 70.36±1.81%, respectively, P<0.05). The percentage of fused embryos that developed to the blastocyst stage was significantly higher in the SSEA-4^+^ cell group compared to the BEF and SSEA-4^−^ cell groups ( P< 0.05).

**Table 3:** Development of cloned bovine embryos in vitro.

**Donor cell**	**No. of oocytes**	**No. of fused embryos (%)**	**No. of cleaved embryos (%)**	**No. of blastocyst (%)**
BEF	253	198(78.19±1.31)^a^	139 (70.36±1.81)^a^	64(32.45±1.47)^a^
SSEA-4^+^	255	187(73.31±1.74)^a^	160(85.62±1.16)^b^	75(40.16±1.12)^b^
SSEA-4^−^	247	183(73.92±1.38)^a^	121(66.32±1.47)^a^	51(27.42±4.57)^c^

Three replicates were performed. The data were shown as Mean±SEM.

^a,b,c^Values with different superscripts within columns are significantly different from each other ( p <0.05).

BEF, bovine embryonic skin fibroblast cells; SSEA-4^+^, stage-specific embryonic antigen 4-positive cells; SSEA-4^−^, SSEA4-negative cells.

### Quality of cloned embryos

**Figure 6:**
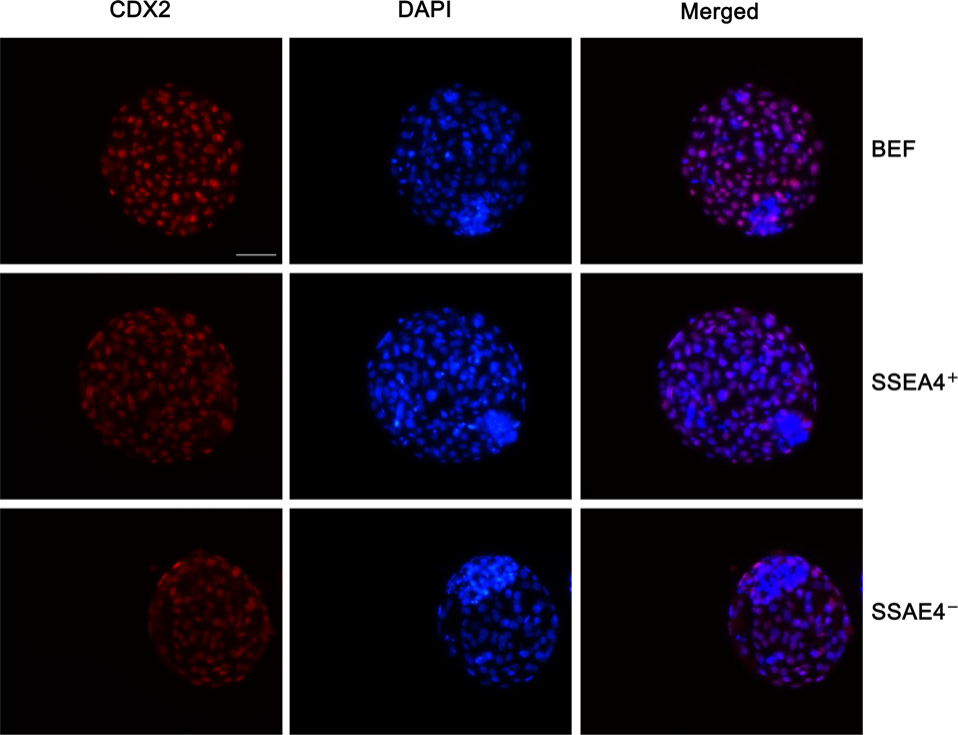
Immunostaining of CDX2 in cloned blastocysts. Day 7 SCNT blastocysts developed from BEF, SSEA-4＋ and SSEA-4－ cells were stained with CDX2 and DAPI. Blastocyst cell numbers were estimated by counting the total number of nuclei using DAPI (blue). The number of trophectoderm (TE) nuclei was estimated using immunostaining for CDX2 (red). Scale bar = 50 μm.

**Figure 7:**
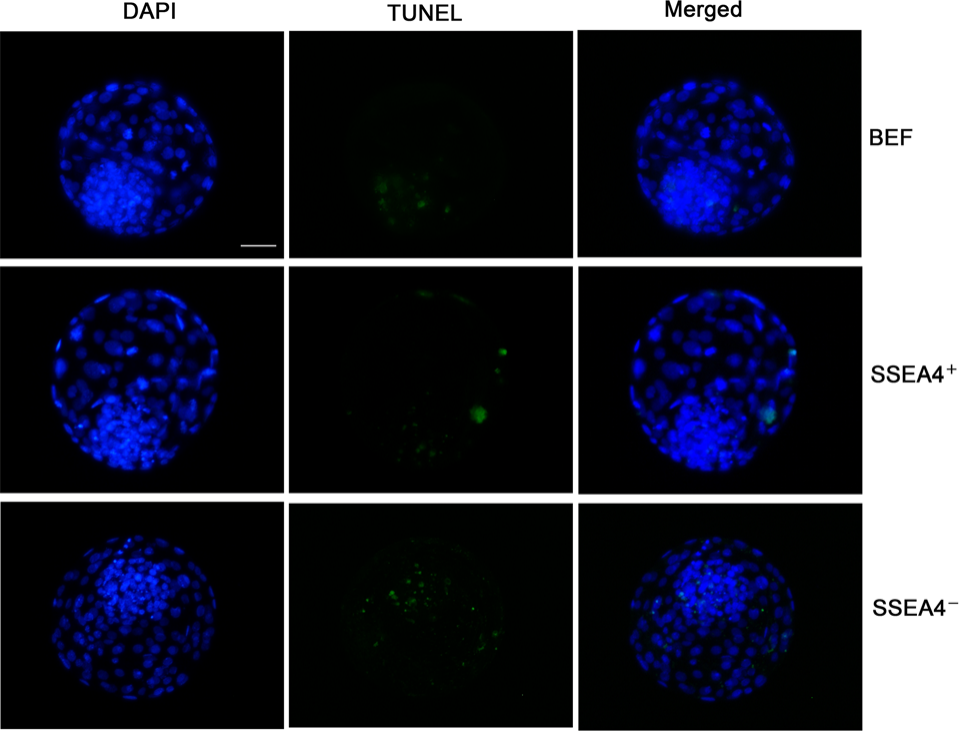
Apoptosis assay in cloned blastocysts. ICM cell number was assessed as the total number of nuclei minus the number of TE nuclei. Apoptotic nuclei were labeled by TUNEL (green), DNA was stained with DAPI (blue). Scale bar = 50 μm.

The quality of cloned blastocysts from BEF, SSEA-4^+^, and SSEA-4^−^ cells was assessed in terms of total cell number, ICM/TE ratio, and apoptosis. Total cell number, TE cell number, ICM cell number and ICM/TE ratio of cloned blastocysts from SSEA-4^+^ cells were all significantly higher as compared to BEF and SSEA-4^−^ cells (Fig. 6; Table 4). There was a significant difference between SSEA-4^+^ and SSEA-4^−^ groups with respect to the rate of blastocyst cell apoptosis. The relative number of apoptotic blastocyst cells was lower in the SSEA-4^+^ cell group compared to the BEF and SSEA-4^−^ cell groups ( P< 0.05) (Fig. 7; Table 5). These results demonstrated that the quality of cloned blastocysts from SSEA-4^+^ cells was higher than those derived from BEF and SSEA-4^−^ cells.

**Table 4:** Total cell number and ratio of ICM/TE of day 7 bovine blastocysts.

**Donor cell**	**No. of blastocysts examined**	**Total no.of cells**	**No. of TE cells**	**No. of ICM cells**	**Ratio of ICM/TE(%)**
BEF	23	107.67±3.20^a^	83.33±2.19^a^	24.67±1.45^a^	29.56±1.16^a^
SSEA-4^+^	26	130.66±2.33^b^	91.67±1.76^b^	42.00±1.53^b^	45.89±2.35^b^
SSEA-4^−^	25	81.33±0.88^c^	53.0±1.15^c^	19.00±1.15^c^	35.78±1.40^c^

The cell numbers in blastocysts were estimated by counting the total number of nuclei using DAPI, and the number of trophectoderm (TE) nuclei was estimated using immunostaining for CDX2. The cell number of the ICM was assessed as the total number of nuclei minus the number of TE nuclei. Three replicates were performed. The data were shown as Mean±SEM.

^a,b,c^Values with different superscripts within columns are significantly different from each other ( p <0.05).

ICM, inner cell mass; TE, trophectoderm. BEF, bovine embryonic skin fibroblast cells; SSEA-4^+^, stage-specific embryonic antigen 4-positive cells; SSEA-4^−^, SSEA4-negative cells.

**Table 5:** Apoptosis of cloned blastocysts.

**Donor cell**	**No. of blastocysts**	**No. of embryos positive for apoptosis (%)^1^**	**No. of apoptotic nuclei per embryos (%)^2^**
BEF	21	17(81.16±2.36)^ab^	3.05±0.43^a^
SSEA-4^+^	24	18(75.33±6.81)^a^	2.67±0.32^b^
SSEA-4^−^	22	19(86.30±8.30)^b^	3.68±0.37^a^

^1^The percentage of embryos containing at least one apoptotic nucleus.

^2^Average number of apoptotic nuclei per embryo.

^a,b^ Values with different superscripts within columns are significantly different.

BEF, bovine embryonic skin fibroblast cells; SSEA-4^+^, stage-specific embryonic antigen 4–positive cells; SSEA-4^−^, SSEA4-negative cells.

## Discussion

SCNT offers a promising strategy for therapeutic cloning, and has potential applications in species preservation, livestock propagation, transgenic research, and disease models. However, despite major efforts in the last decade to improve this technology, efficiency still remains in the single-digit percentage range [1]. In the process of SCNT, the nucleus of a somatic cell is transplanted into an enucleated oocyte where it is then reprogrammed by the oocyte microenvironment. It has been suggested that less differentiated cells may be more amenable to nuclear reprogramming than terminally differentiated cells [4], and may therefore provide a better source of candidate donor cells for SCNT. Pluripotent stem cells are also regarded as a potential source of cells that can be genetically manipulated easily to produce transgenic and genetically modified domestic animals. For example, bovine ES-like cells were used as the source of nuclei for NT to produce cloned cattle with a higher frequency of full term pregnancies than those achieved using somatic cells [33]. Additional studies have shown that cloned embryos derived from undifferentiated bovine MSCs gave rise to consistently higher rates of pre-implantation development as compared to those derived from adult fibroblasts [34]. However, due to the lack of stable pluripotent bovine stem cell lines, there remains a critical need to improve the technology of SCNT in order to systematically produce transgenic and genetically modified cattle. One method of enhancing SCNT efficiency is by optimizing the cell type for nuclear transfer.

Many adult tissues contain a heterogeneous population of cells that includes stem cells, progenitor cells, and terminally differentiated cells. In recent years, several studies have demonstrated that a subpopulation of stem cells exists within both human fibroblasts and bone marrow stromal cells [23, 24, 35]. SSEA-4 is a cell surface marker detected in pluripotent cells, that has previously been used as a marker to isolate novel stem cell subpopulations from human bone marrow [15, 16], human pancreas [17], human dermis [18] and other tissues [19–21]. Furthermore, a rare population of novel pluripotent stem cells known as very small embryonic-like stem cells (VSELs) have been isolated from cord blood and bone marrow tissues through different isolation procedures [36–40]. Because the population of multipotent stem cell from human fibroblastes showed enhanced efficiency in reprogramming, the multipotent stem cells from bovine skin fibroblastes were used as SCNT donor cells.

In this study, we fully characterized a population of stem cells expressing SSEA-4 derived from fibroblasts isolated from bovine embryonic skin. We isolated a subpopulation of bovine embryonic skin fibroblasts expressing SSEA-4 by MACS (2–3%). It was a major challenge to maintain the undifferentiated state of SSEA-4^+^ cells because that little is known about culture conditions that support SSEA-4^+^ cells proliferation in vitro. Previous reports have shown that stem cells were isolated from skin tissue via selective growth of floating spheres [7, 23, 25, 27]. Therefore, we maintained SSEA-4^+^ cells in suspension culture using optimized medium containing EGF and bFGF in order to retain the ability of cells to self-renew, as described in a previous study [25]. The SSEA-4^+^ cells were able to form cell clusters in suspension culture. SSEA-4^+^ cells isolated by MACS were small and round with diameters of 8–10 μm (smaller than skin fibroblasts), had a high nuclear/cytoplasmic ratio, stained positive for AP. They were also consistently positive for the pluripotent stem cell markers NANOG, OCT4, SOX2, SSEA-4 and CD105. Upon continued culture, SSEA-4^+^ cells expressed increasing levels of these markers. While the thses cells demonstrated the ability to self-renew and give rise to cells of all three germ layers, in contrast to pluripotent stem cells, they do not form teratomas. The lack of teratoma formation implies that the isolated SSEA-4^+^ cells were multipotent stem cells. Multipotent stem cells isolated from human adult and fetal dermis showed mesenchymal characteristics [41]. These multipotent bovine SSEA-4^+^ cells also exhibited features of mesenchymal stem cells. Compared with MSCs derived from human dermis [18], the SSEA-4^+^ cells expressed mesenchymal markers, CD166, CD105, CD29, CD44 and were able to differentiate into adipocytes, osteocytes, and chondrocytes. These results showed the SSEA-4^+^ cells have partial characteristics of dermis-derived MSCs. However, the morphology of undifferentiated SSEA-4^+^ cells were different from MSCs. By contrast, the differentiated SSEA-4^+^ cells were morphologically similar to MSCs. Toma et.al [7] reported that skin-derived precursors (SKPs) isolated from mouse skin could differentiate into neurons, glia, smooth muscle cells and adipocytes. SKPs expressed Nestin and were distinct from mesenchymal stem cells. Overall, our results showed that SSEA-4^+^ cells are a rare population of stem cells that exist in skin tissue that exhibit multiple characteristics of multipotency.

It has been suggested that undifferentiated cells may be more amenable to reprogramming or require less reprogramming following nuclear transfer than differentiated cells [4, 6]. Therefore, in the present study, we examined whether multipotent SSEA-4^+^ cells isolated from fetal skin can be used to improve somatic nuclear reprogramming and the development of cloned bovine embryos in vitro. We found that cloned embryos from SSEA-4^+^ cells had significantly better indicators of quality than the other cells tested. The cell number, especially the ratio of ICM to TE cells, is one of the criteria for assessment of blastocyst quality [42]. We counted total blastomere cell numbers, TE and the estimated ICM cell numbers and measured the ratio of ICM to TE in blastocysts. Interestingly, cloning using SSEA-4^+^ cells increased the total number of cells, as well as the number of ICM cells in SCNT blastocysts. The ratio of ICM to TE was also increased in the SSEA-4^+^ cells group. Apoptosis is another criterion for evaluating blastocyst quality, as it eliminates cells with nuclear or chromosomal abnormalities [43]. Apoptosis can be detected in embryos after the 8-cell stage in bovine embryos [44]. High rates of apoptosis in SCNT blastocysts are correlated with a decrease in the total cell number [45]. In this study, the number of apoptotic cells was significantly lower in the blastocysts derived from SSEA-4^+^ cells as compared to those derived from BEF and SSEA-4^−^ cells. These results demonstrated that SSEA-4^+^ cells improve the quality of SCNT embryos by reducing cell death in the embryos. Therefore, SSEA-4^+^ cells can serve as an excellent candidate donor cells. In the process of SCNT, the nucleus of a somatic cell is transplanted into an enucleated oocyte. The somatic cell nucleus is reprogrammed in the environment of the oocyte. In practice, we found that the donor cells with long cilia were easily reprogramed after NT and the cloned embryos had higher developmental capacity (data not shown). Aside from their stem-like qualities, the SSEA-4^+^ cells isolated in this study also displayed a rough surface with a long flagellum. Primary cilia of cells play an important role in transmiting signals to the interior of cells. Cilia sense a wide variety of extracellular signals and transduce them into decisions regarding proliferation, polarity, nerve growth, differentiation, or tissue maintenance [46].

In conclusion, our study demonstrates that SSEA-4^+^ cells derived from bovine fetal skin fibroblasts are a novel homogeneous subpopulation expressing pluripotent stem cell markers. Furthermore, SSEA-4^+^ cells provide an excellent candidate for nuclear donor cells which can enhance the development and the quality of SCNT cloned embryos in vitro.
